# Systems biology successes and areas for opportunity in prostate cancer

**DOI:** 10.1530/ERC-25-0067

**Published:** 2025-08-20

**Authors:** Michael V Orman, Laura S Graham, Scott D Cramer, James C Costello

**Affiliations:** ^1^ University of Colorado Anschutz Medical Campus, Department of Pharmacology, Aurora, Colorado, USA; ^2^ University of Colorado Anschutz Medical Campus, Department of Medical Oncology, Aurora, Colorado, USA; ^3^ University of Colorado Anschutz Medical Campus, University of Colorado Comprehensive Cancer Center, Aurora, Colorado, USA

**Keywords:** systems biology, prostate cancer, adaptive therapy, molecular landscape, tumor evolution

## Abstract

Systems biology approaches have been applied to prostate cancer to model how individual cellular and molecular components interact to influence cancer development, progression, and treatment responses. The integration of multi-omic experimental data with computational models has provided insights into the molecular characteristics of prostate cancer and emerging treatment strategies that have the potential to improve patient outcomes. Here, we highlight recent advancements that have emerged from systems modeling in prostate cancer. These include descriptions of the molecular landscape of prostate cancer and how genomic alterations inform computational models of disease progression, how evolutionary processes give rise to mechanisms of therapeutic resistance, and the development of innovative treatment strategies such as adaptive therapy. We also highlight current challenges in prostate cancer that can be addressed through systems biology approaches. These include tumor heterogeneity, poor immunotherapy response, a paucity of experimental model systems, and the ongoing translation of computational models for clinical decision making. Leveraging systems biology approaches has the potential to lead to a better understanding of the disease and better patient outcomes in the treatment of prostate cancer.

## Introduction

Cancer cells and tumors are part of, and function within, systems, whether that is at the molecular level of signaling networks, within the local microenvironment of the organ where the cancer arose, or more broadly within the individual person. Cancer systems biology broadly aims to study how individual elements or components come together to function as a system to drive cancer development, progression, metastasis, and response to treatment (1, 2, 3). The components of the system can be molecules, which together give rise to signaling networks. The components can also be individual cells and cell types that can combine to drive cancer phenotypes in the tumor microenvironment. These systems can be perturbed in many ways, whether that is from somatic alterations or from pharmacological agents. The properties and phenotypes that emerge from these systems are what cancer systems biology aims to model (4), and in order to study these phenomena, we must have methods to measure the components of the system.

The rise of multi-omic data modalities and many sophisticated offshoots have given us the means to measure molecular components of tumors at the bulk and single-cell level. In addition, technological advances in quantification at the single-cell level have given us new methods to measure the cellular composition of tumors (5). The integration of these two approaches has more recently given us the ability to combine cell types with the molecular features in a spatially resolved manner within the tumor microenvironment (6). Prostate cancer has been at the forefront of systems biology modeling with successful basic research and early-stage clinical applications (7, 8, 9, 10, 11, 12, 13, 14); however, prostate cancer has not had the same success at characterizing molecular mechanisms of tumor development and novel therapeutic targets as other cancers. Here, we will highlight the overall successes in cancer systems biology in the context of prostate cancer and also discuss where opportunities exist for systems biology approaches to continue to advance research and clinical translation in prostate cancer.

There are many features of prostate cancer that make it an ideal cancer type to apply systems modeling approaches. First, because it is the most common cancer in men in the United States and the second most common cancer in men worldwide (15), there are a large number of prostate cancer samples collected across many diverse clinical settings. As a result, prostate cancer is one of the most commonly sampled tumor types for multi-omics data modalities, providing researchers with a large number of molecular measurements (16, 17, 18, 19, 20, 21, 22, 23, 24, 25, 26). Second, we have strong evidence that molecular features of prostate cancer can be used to predict treatment outcomes and disease progression in multiple settings. There are commercially available molecular diagnostics for these purposes, such as Decipher (27), Oncotype DX (28), and Prolaris (29). Knowing that the molecular data of prostate cancer contain positive predictive information about patient outcomes supports further model development to understand the mechanisms of disease development and treatment response. Third, after establishing baseline levels, PSA can be easily measured using a blood draw and is a strong indicator of disease progression or regression, especially in the early course of the disease. Accordingly, PSA can be used as a proxy for tumor response to treatment and is a unique biomarker to prostate cancer. Fourth, and for almost all prostate cancers, we know the main causal driver of disease development and treatment resistance. The androgen receptor and androgen signaling are critical in almost all aspects of prostate cancer and allow us to focus model development on the most relevant molecular elements to the disease.

**Figure 1:**
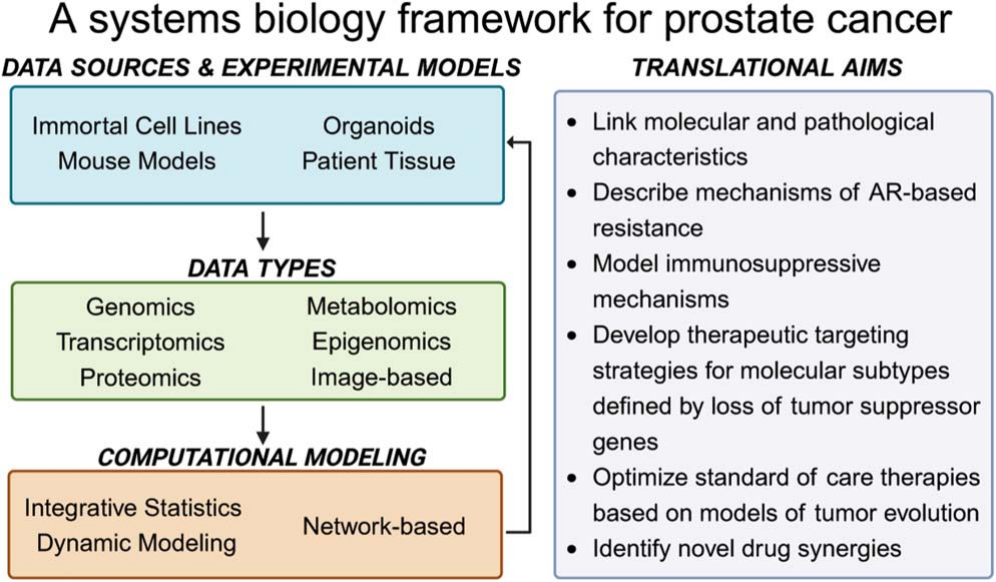
Systems biology framework for conducting prostate cancer research. Experimental models and patient-derived samples provide information in the form of multi-omics datasets, which are analyzed by a wide range of computational methods. Predictions generated by computational modeling are formed into testable experimental hypotheses that can be evaluated in these same model systems.

Because of these modeling advantages in prostate cancer, we have seen several important advancements in basic science and emerging treatment strategies. We highlight three areas where systems modeling and data integration have led to novel insights and strategies in studying and treating prostate cancer. These include large multi-omic data generation and integration efforts to characterize the molecular landscape of prostate cancer, the modeling of tumor evolution to gain insights into tumor dynamics that drive prostate cancer development, and how adaptive therapy anchored on PSA levels has led to better outcomes in the metastatic castration resistant prostate cancer (mCRPC) setting (Fig. 1). While these results are exciting and demonstrate the promise of systems biology approaches in prostate cancer, there remain a number of challenges. Accordingly, we conclude by discussing these challenges, including tumor heterogeneity, the immune microenvironment, tumor suppressors as drivers of prostate cancer, and experimental model systems. These challenges highlight the opportunities for future growth and development of systems modeling in prostate cancer.

## The molecular landscape of prostate cancer is defined by loss of tumor suppressor genes and chromosomal instability

**Figure 2:**
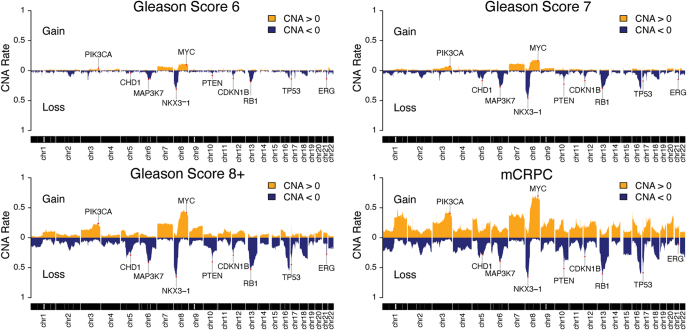
The CNA landscape of primary and metastatic castration-resistant prostate cancer (mCRPC). Analysis of CNA rates in primary tumors is stratified according to the Gleason scoring system. Copy number gains are shown in orange and copy number losses are shown in blue.

The molecular landscape of primary prostate cancer has been well-characterized through numerous tumor profiling studies published over the past two decades (16, 17, 18, 19, 20, 21, 22, 23, 25, 26, 30). These studies have found that the prostate cancer genome is characterized primarily by structural variation in the form of gene fusion events and copy number alterations (CNAs). In primary tumors, rearrangements involving ETS-family transcription factors and androgen-regulated genomic elements are found in a large percentage of cases and contribute to oncogenic overexpression of ETS-family fusion transcripts (30). The most common example is the fusion of the androgen-responsive *TMPRSS2* gene promoter to the 5′ end of the *ERG* gene (30). The *TMPRSS2* and *ERG* fusion occurs through translocation and deletion events and the prevalence of this alteration varies between 16 and 50% depending on patient ancestry (31). Additional ETS-family transcription factors that undergo fusion events include *ETV1, ETV4,* and *FLI1* (26). CNAs are abundant in primary prostate cancer and have prognostic value when predicting biochemical recurrence and metastasis-free survival (25, 32). Combined analysis of over 900 primary tumors across five independent profiling cohorts was performed using a harmonized prostate cancer data resource (24). This analysis confirmed several tumor suppressor genes playing a role in disease progression that are found in regions of heavy genomic loss spanning 5q11-5q23 (*CHD1*), 6q12-6q22 (*MAP3K7*), 8p11-8p23 (*NKX3-1*), 10q22-10q26 (*PTEN*), and 13q12-13q24 (*RB1*) (33). As shown in Fig. 2, further stratification of primary tumors based on the Gleason scoring system show that these regions of copy number loss are enriched throughout the primary prostate cancer disease spectrum. *NKX3-1* is the most commonly lost tumor suppressor gene in primary tumors, with its deletion found in about half of patients, and is among the first alterations to occur in prostate cancer development (21, 34). Loss of *NKX3-1* is associated with PIN in human samples and also drives PIN development in the mouse prostate (35). *MAP3K7* and *RB1* are also some of the most commonly lost tumor suppressor genes in primary tumors at roughly 30 and 34%, respectively (33). Loss of *MAP3K7* expression is associated with biochemical recurrence and aggressive Gleason scores, and drives prostate tumorigenesis in mice (36, 37). Interestingly, *RB1* loss has been identified as an alteration occurring early in prostate cancer development that drives clinical aggression through subclonal diversification (34). Deletion mapping studies have found loss of *PTEN* in 26% of prostate tumors and functional analysis in murine models has shown that homozygous *PTEN* loss is sufficient to drive disease progression from early stages of prostate cancer formation to metastatic disease (38, 39). In addition, loss of the chromatin-remodeling protein *CHD1*, in about 20% of tumors, reprograms the *AR* cistrome into an oncogenic state and contributes to tumor progression (40). Amplifications are less common than gene deletion in primary tumors. Nonetheless, the most amplified regions observed in primary tumors span chromosomes 3q22-3q26, 8q11-8q24, and chromosome 7 (Fig. 2) (33). These regions harbor known oncogenes driving prostate cancer, including *MYC, NCOA2, PIK3CA,* and *ETV1*, and also implicate genes with recently-discovered oncogenic functions such as *PLXNA1* (25, 26).

In addition to extensive structural variation, the genomic instability of prostate cancer gives rise to low-frequency mutations that impact prostate cancer progression. Initial efforts to profile mutations in 112 treatment-naive primary prostate samples with matched normal tissue identified 12 significant mutations across several cancer genes, including *TP53, PTEN, PIK3CA,* and *CDKN1B* (20). Novel mutations were also identified in *FOXA1, MED12,* and *SPOP* (20). *SPOP* was the most highly mutated gene found in 13% of samples and follow-up work has now solidified *SPOP* loss-of-function mutations as robust drivers of prostate cancer (20, 41, 42). Mutation profiling by TCGA has confirmed the prevalence of *SPOP* and *FOXA1* mutations and discovered *IDH1* as a recurrently mutated gene that defines primary tumors (26). Larger-scale genomic characterization pooling over 1,000 primary and metastatic prostate cancer samples found 97 significantly mutated genes, with groups of these genes functioning in WNT, spliceosome, proteasomal degradation, and epigenetic signaling pathways (18).

The molecular landscape of mCRPC has also been well-defined in recent years. Profiling studies show that mCRPC is characterized by a high alteration and mutation burden, and increased alteration frequency to potent disease drivers including *AR* and the tumor suppressor genes *TP53, RB1,* and *PTEN* (Fig. 2) (16, 19, 25, 26, 43). *AR* amplification is observed in roughly half of mCRPC cases and is mutated in about 10% of cases (16, 43). *TP53* is the most highly mutated gene in mCRPC observed in about 30% of cases with almost all of these mutations predicted as oncogenic (16, 43). *PTEN* and *RB1* are also significantly mutated in mCRPC; however, these genes are largely enriched with copy number loss resulting in deletion frequencies of about 30 and 15%, respectively (16, 43). Common mCRPC alterations have also been grouped at the pathway level to identify dysregulated signaling pathways contributing to aggressiveness. In particular, alterations in genes regulating AR, PI3K/Akt, WNT, DNA-repair, cell cycle, and epigenetic signaling pathways are commonly observed in the mCRPC setting (16, 18, 19). Several of the genomic alterations that are enriched in mCRPC have been used to define the neuroendocrine prostate cancer (NEPC) subtype. NEPC is largely found in metastases late in disease development as an adaptive response to inhibiting the AR signaling axis with ARSI therapies (44). Characterization of NEPC biopsies has found loss of AR expression and its downstream targets combined with the loss of tumor suppressors including TP53, RB1, and PTEN (45). Another defining feature of NEPC is the induced expression of neuroendocrine markers such as synaptophysin (SYP), chromogranin A (CHGA), CD56, neuron-specific enolase (NSE), and bombesin (BRS3) (45). Rigorous phenotypic characterization of mCRPC tumors and PDX models has further reinforced the importance of AR and neuroendocrine features in classifying advanced prostate cancer. This work defined five subclasses of mCRPC: uniformly AR-expressing tumors with no neuroendocrine features (ARPC), tumors with weak or diffuse AR expression and no neuroendocrine features (AR-low prostate cancer, or ARLPC), tumors expressing both AR and neuroendocrine markers (amphicrine prostate cancer or AMPC), AR-negative tumors with neuroendocrine features (small cell/neuroendocrine prostate cancer, or SCNPC), and tumors that exhibit neither AR expression nor small-cell neuroendocrine features (double-negative prostate cancer or DNPC) (46).

The alterations defining prostate cancer do not occur in isolation but rather co-occur with one another throughout the disease spectrum. A number of research groups have addressed the topic of multigenic molecular subtypes to understand how combinations of prostate cancer alterations may cooperate to drive aggressiveness. Dual loss of *NKX3-1* and *PTEN* is a well-characterized example of coordinating alterations in prostate cancer. In a tissue recombination system with serial transplantation, mice with heterozygous loss of *NKX3-1* and *PTEN* developed high-grade PIN with invasive adenocarcinoma in 84% of cases and lymph node metastasis in 25% of cases (47). Mice with only *NKX3-1* loss showed neither of these aggressive phenotypes and mice with only *PTEN* loss showed invasive adenocarcinoma in 54% of cases and no lymph node metastasis, indicating heterozygous loss of *NKX3-1* or *PTEN* alone is insufficient to drive metastasis (47). Mechanistically, *PTEN* and *NKX3-1* interact in the nucleus where *PTEN* functions as an *NKX3-1* phosphatase to protect *NKX3-1* from degradation (48). *PTEN* loss has also been shown to drive aggressive prostate cancer in combination with *RB1* and *TP53* loss (49). In conditional knockout models, *Rb1* loss is sufficient to drive metastasis and lineage plasticity following tumor formation driven by *Pten* loss. Additional *Trp53* loss was modeled in these double-knockout mice, resulting in resistance to antiandrogen therapy (49). *TMPRSS2–ERG* fusion and *PTEN* loss is another example of coordinating interactions in prostate cancer. Aberrant *ERG* overexpression in the mouse prostate did not have a significant phenotype, while heterozygous loss of *PTEN* results in high-grade PIN by 8 months of age (50, 51). However, when mice were engineered to have prostatic *ERG* overexpression on a heterozygous *Pten* background, this resulted in multifocal prostatic adenocarcinoma by 6 months of age (51). Further characterization showed that ERG promotes transcriptional upregulation of *CXCR4* and *ADAMTS1*, both of which regulate cell migration (51). Another example is the co-loss of *MAP3K7* and *CHD1*, two tumor suppressor genes, which coordinate to drive disease aggressiveness. In a developmental model based on tissue recombination, co-suppression of *CHD1* and *MAP3K7* drove the formation of high-grade PIN and intraductal carcinoma *in vivo* (52). These pathological features coincided with sporadic clusters of *SYP* expression, indicating neuroendocrine differentiation (52). Mechanistic analysis of this subtype has shown that the co-suppression of *CHD1* and *MAP3K7* cooperatively boosts AR signaling by increasing AR nuclear translocation and chromatin binding (37).

## The molecular landscape of prostate cancer in systems biology modeling

Defining the molecular landscape of prostate cancer is a critical first step in understanding how these alterations contribute to disease development, progression, and therapy response. Systems biology approaches leverage this information to model how alterations can affect molecular pathways and processes driving cancer phenotypes. Montagud *et al.* leveraged a Boolean model and integrated 498 prostate cancer samples sourced from TCGA along with cell line information sourced from the Genomics of Drug Sensitivity in Cancer (GDSC) database (53). A cancer signaling Boolean network was adapted from Fumiã *et al.* to further capture the known regulatory relationships between common prostate cancer alterations (54). *NKX3-1* deletion was modeled by deleting the node required for the activation of TMPRSS2:ERG by AR. *SPOP* mutations were modeled by removing the node that triggers apoptosis in the presence of cell death factors. Invasion was encoded by the activation of a combination of *CHD2, SMAD,* and *EZH2*. Progression to bone metastasis was activated by a set of invasion and migration modules combined with the activation of ERG, FLI1, ETV1, or ETV4 fusions (10). The modified networks were used as patient and cell line-specific models to investigate mechanisms of tumorigenesis and tumor growth. Patient-specific model simulations validated increased proliferation and decreased apoptosis in models trained on the high-grade samples compared to the low-grade ones. This approach was applied to LNCaP cells to generate a cell line-specific model that identified druggable targets, including HSP90 and PI3K (10).

Using a different modeling approach, Kuipers *et al.* trained a Bayesian network using pan-cancer mutational profiles spanning 22 different cancer types and 201 non-silent mutations (55). This dataset contained 498 prostate adenocarcinoma samples harboring mutations in canonical prostate cancer genes, including *TP53, RB1, PTEN, BRCA PIK3CA,* and *SPOP*. Across all cancer types, various mutational dependencies in genes such as *TP53* and *MLL2* that encompassed multiple cancer types were revealed. Dependencies that were specifically associated with prostate cancer included co-occurring mutations between *KEAP1* and *JUB, SPOP* and *EME2,* and *EME2* and *ARIDA1* (55). Features extracted from this modeling approach were further used to define subgroups and provide an alternate stratification scheme for predicting prostate cancer prognosis based solely on mutational status (55). In another example of Bayesian modeling, Blatti *et al.* sourced transcriptional data from mCRPC patients classified as either responders or non-responders following abiraterone and prednisone dual-therapy (56). These transcriptional data were used to train a sparse Bayesian network followed by differential network analysis to identify rewiring of the model that was associated with castration resistance. The approach, named TraRe, identified several well-known transcription factors regulating key network modules, the most notable being an immune response module driven by *GATA1, CEBPE, KLF1,* and *MYB* transcriptional activity. TraRe predictions were tested using abiraterone-sensitive prostate cancer cell lines to confirm the function of *ELK3, MXD1,* and *MYB* in promoting castration-resistant growth (56).

Overall, the molecular landscape of prostate cancer provides the features needed to develop and test systems biology models, including tumor initiation, growth, and the development of castration resistance. The accuracy of the models depends on a robust characterization of the molecular features associated with aggressive prostate cancer. These computational models have generated novel hypotheses that can then be tested using experimental models to establish causal variables in the etiology of prostate cancer.

## The evolution of prostate cancer follows several trajectories

Prostate cancer progression can be modeled as an evolutionary process by which the accumulation of genomic alterations during the lifetime of the tumor promotes development, progression, and metastasis. Work in this area has leveraged whole genome sequencing (WGS) data to generate CNA and single nucleotide variant (SNV) profiles, which can be used to reconstruct the temporal sequence of alterations occurring over tumor progression. Analysis of CNA clonality has shown *NKX3-1* deletion, *RB1* deletion, *FOXP1* deletion, and *ERG* rearrangement are the earliest forms of structural variation occurring in prostate cancer evolution (34, 57). Occurring alongside these structural variants, analysis of SNV clonality identified *ATM, FOXA1, SPOP* and *TP53* mutations as early evolutionary events (21, 34). These data have been used to propose evolutionary trajectories of prostate cancer tumors. Baca *et al.* proposed a consensus path of progression that starts with deletions in *NKX3-1*, *FOXP1*, and *TMPRSS2-ERG* (21). From there, alterations in *TP53* and *CDKN1B* promote tumor growth and are eventually followed by loss of *PTEN* to drive prostate cancer aggressiveness (21). Espiritu *et al.* proposed a different evolutionary trajectory defined by early loss of *NKX3-1* and *RB1* and SNVs in *FOXA1* and *ATM* (34). These initiating alterations are then followed by driver SNVs in *SPOP* and *TP53*; afterward, subclonal driver amplifications accumulate in genes including *MTOR*, *TCS1, TCS2, MAF, BAD, BID*, and *BAK1* (34). Additional work assessing CNA clonality described distinct phylogenies of alterations occurring in *ETS* transcription factor fusion positive (*ETS+*) and *ETS* fusion negative (*ETS*−) prostate tumors. *ETS*+ tumors were defined by an early *TMPRSS2*–*ERG* fusion, followed by early gains of chr8q, and then subsequent homozygous deletion to chr5 (*PPAP2A*, *PDE4D*, *MAP3K1* and *IL6ST*) and chr10 (*PTEN*) (58). Early homozygous deletions at chr5 (*CHD1* and *RGMB*) and chr13 (*RB1, BRCA2,* and *FOXO1*) defined *ETS−* tumors, which were then followed by deletions to chr2, gains to chr3, and finally gain across the entirety of chr7 (58). This work also analyzed SNVs and found that 84 were clonal and 22 were subclonal, with frequent *SPOP* mutations that were exclusive to *ETS+* tumors (58). More recent work using WGS proposed a novel evolutionary model, referred to as evotypes, of primary prostate cancer (59). This analysis defined two evolutionary subclasses of tumors, named the canonical evotype and the alternative evotype. Canonical evotype tumors were associated with frequent early *ETS* fusions and less frequent early deletions to loci harboring *PTEN, TP53, RB1,* and *CDH1* (59). Alternative evotype tumors were defined by very early, low-frequency *SPOP* mutations and followed by abundant early-stage *MAP3K7, CHD1,* and *RB1* deletions (59). Consistent with previous work, *NKX3-1* deletion was found as an early alteration at high rates in both the canonical and alternative evotypes (59). Evolutionary models based on gene expression have also been developed to track tumor evolution within subtypes. This work showed that *TMPRSS2*–*ERG* fusions and *SPOP* mutations are clonal while *PTEN* and *CHD1* deletions have subclonal fractions (60). From this, two alteration trajectories have been proposed, including an *ERG* fusion to *PTEN* deletion path and a *SPOP* mutation to *CHD1* deletion path, with each of these trajectories displaying distinct clinicopathologic characteristics (60).

Given the abundance of CNA data available from multiple independent profiling studies in prostate cancer, we generated a CNA landscape stratified by Gleason grade to summarize the key alterations in early-stage disease and show their increasing prevalence over the course of tumor evolution (Fig. 2). In concordance with findings from studies focusing on tumor evolution, our analysis of CNAs clearly identifies *NKX3-1, RB1, MAP3K7, CDKN1B, TP53,* and *ERG* copy number losses as strongly enriched CNAs observed in the earliest stages of prostate cancer (Fig. 2). We also confirm copy number gains to chromosome 7 and chromosome 8q as early-stage alterations in prostate cancer development (Fig. 2). Future efforts to model prostate cancer evolution will benefit from including these alterations as initiating or early-stage events when designing and evaluating systems biology models.

## Adaptive therapy directly integrates computational modeling into treatment regimens

Paradigms of clinical care of prostate cancer can be in conflict with how the disease develops and evolves in response to treatment. In the metastatic setting, treatment almost always starts with androgen deprivation therapy (ADT). Treatment is often effective at first, but the cancer eventually progresses past ADT into the metastatic castration-resistant setting. Attempts were made to treat prostate cancer in the metastatic (61) and non-metastatic setting (62, 63) using intermittent ADT compared to continuous treatment (combinations of luteinizing hormone-releasing hormone (LHRH) analogs and antiandrogens). Intermittent ADT was hypothesized to potentially reduce selective evolutionary pressure toward treatment resistance while also allowing patients meaningful treatment breaks designed to improve the quality of life. The results showed little to no difference in clinical cancer outcomes using intermittent ADT. In these clinical trials, patients underwent an induction phase of ADT and then proceeded with a fixed schedule of intermittent or continuous ADT. While the trial design offered a new treatment schedule for patients, the same schedule was applied to all patients, which did not allow for adjusting the schedule or dosing based on an individual’s response to treatment.

Gatenby and colleagues approached treatment resistance from an ecological perspective and developed computational models of how prostate cancer tumor cells interact given environmental constraints and cellular interactions (8, 9, 64, 65, 66, 67, 68). The models derived from these principles showed that if certain dominant tumor cell populations were selectively removed through simulated drug treatment, then other populations that were suppressed by the dominant population were allowed to expand. Termed competitive release, these suppressed populations were both resistant to the treatment and more aggressive if allowed to expand. Thus, the treatment itself selects for tumor cells that drive treatment resistance and aggressiveness of disease, ultimately leading to death due to mCRPC.

The models by Gatenby and colleagues led to clinical trials of an alternative treatment approach where metastatic prostate cancer was treated with AR targeted therapy, but with early treatment breaks to allow for persistence of sensitive cells. In contrast to the fixed schedule with prior intermittent therapy trials, in the pilot study of men with mCRPC treated with ADT and the AR signaling inhibitor (ARSI) abiraterone, abiraterone was paused if PSA levels reached 50% of the baseline level and then restarted when PSA levels returned back or exceeded pretreatment baseline. ADT was maintained throughout. This approach, termed adaptive therapy, is customizable to every patient. A total of 16 men were in the adaptive therapy arm and 11 men in the standard of care, control arm. Men receiving adaptive therapy showed a radiographic progression-free survival of 30.4 months compared to 14.3 months and a median overall survival of 58.5 months compared to 31.3 months in the control arm (67, 69). Both results were highly statistically significant and men receiving adaptive therapy received fewer treatments, resulting in an estimated $70,000 reduction in cost per patient per year (70). Another theoretical benefit of adaptive therapy is the opportunity for patients to have treatment holidays which may improve patient reported outcomes, including health-related quality of life, sexual functioning, and emotional and physical well-being. A phase 1b study in men with metastatic castration-sensitive prostate cancer (mCSPC) was performed by cycling a LHRH analog and an ARSI (i.e., abiraterone, enzalutamide, or apalutamide). Treatment was paused if PSA levels decreased by 75% after 12–16 weeks of therapy and restarted at the time of radiographic or PSA progression (69). This study demonstrated the feasibility of this approach; however, both trials are limited by their small sample size and lack of prospective randomization. Further validation is needed before this approach becomes more widely adopted.

The use of mathematical models was critical in the development of adaptive therapy (65, 71, 72, 73). In prostate cancer, these models continue to evolve to include additional treatment regimens, such as docetaxel in the mCRPC setting (68, 74). In addition, modeling approaches can consider the impact of spatial architecture on treatment response (75), along with how the treatment shapes the local tumor microenvironment (76, 77). In addition, the adaptive therapy paradigm has been expanded to include not only treatment cycling, but also dose modulation in response to treatment. Dose modulation is responsive to tumor size with an increase in dose if there is a tumor size increase over 20% and a decrease in dose if there is a decrease by 20% (8, 65, 78). There is active research to expand this approach to other cancer types, including in melanoma (79, 80, 81), breast (82, 83), colorectal (84), and ovarian cancers (85). Dose modulation may be challenging to apply to metastatic prostate cancer, which often has bone-predominant disease, where assessment of tumor volume is difficult. The adaptive therapy paradigm requires more research and clinical trials to evaluate its overall effectiveness, although the fact that the first clinical trials were performed in prostate cancer demonstrates that systems biology approaches can be effective in this cancer type.

## Challenges that limit systems modeling in prostate cancer

While the previous examples demonstrate that systems biology approaches and multi-omics have led to important basic research and clinical insights, there are some fundamental challenges that prostate cancer researchers face, including tumor heterogeneity, the lack of a strong immune contribution to the prostate cancer microenvironment for translation of immune checkpoint inhibitors, the observation that prostate cancer is driven by loss of tumor suppressors, and the lack of cell culture models that accurately represent the diversity of genomic alterations and primary tumor pathology.

Prostate tumor heterogeneity can take many forms, including, pathological, genetic, the tumor microenvironment, and the secretome. Heterogeneity can be between two tumors from different individuals (i.e., different genetic subtypes or pathological grades or patterning) or between different tumors within the same individual. Unlike many tumor types, it is not uncommon to find multiple, independent foci in an individual’s prostate (86). The Gleason scoring system identifies the two most dominant patterns that are observed by the scoring pathologist, regardless of how many patterns or foci of tumor are observed. The index tumor is generally considered the clinically actionable tumor with other tumors considered incidental (86). Boutros *et al.* examined CNAs, SNVs, and WGS data in index tumors of intermediate risk (Gleason 7) (87). The data demonstrated that foci can arise either from a common cell of origin or from independent clones (87). The foci derived from independent foci can represent distinct cancers that may require different therapeutic strategies. One tumor had nine independent foci sampled for WGS. Two Gleason 7 foci (including the index focus) had a *PIK3CA* mutation and loss of *TP53*. The *PIK3CA* mutation was predicted to make AKT inhibition an actionable therapeutic strategy. The other seven foci lacked this mutation. In contrast, four of the other foci had loss of *BRCA2* but retained *TP53*. The approach to therapy in a patient with such heterogeneous individual foci is a major challenge that will require longitudinal sampling and systems modeling. In mCRPC, intertumoral heterogeneity can be present between metastatic sites, with subclonal genetic diversity conferring the ability for treatment resistant clones to be selected (88). On top of these findings, treatment also contributes to subclone selection, adding additional complexity to the modeling of prostate cancer tumor heterogeneity (89).

Prostate cancer is characterized as a ‘cold’ tumor, meaning the tumor microenvironment in the prostate tends to be immunosuppressive and show little to no immune activation (90). While there have been positive associations made with immune cell populations, patient outcomes, and treatment response, the associations are in a minority of the population of prostate cancer patients. The predictive value of immune-related signatures is poor with only a small subset of patients (e.g., CDK12 mutations, mismatch repair deficient, and high microsatellite instability) benefiting from immune checkpoint inhibitors (91, 92, 93, 94). The benefits of the immune system in contributing to disease progression in hot vs cold tumors was first described in colorectal cancer and showed a 2-year relapse rate of 10 vs 80%, respectively (95). Subsequent studies have validated these findings (96, 97), with new ways to score tumors on their likelihood of response to immune checkpoint inhibitors based on immune cell infiltration into the tumor microenvironment (98). More recent work has shown efficacy of turning cold tumors into T-cell inflamed tumors, resulting in stronger and more durable response with immune checkpoint inhibitors (99, 100, 101). In prostate cancer, there are several mechanisms that drive an immunosuppressive microenvironment, including the function of regulatory T cells, tumor-associated macrophages, and myeloid-derived suppressor cells, interactions with stromal cells, and production of adenosine from prostatic acid phosphatase (102). These mechanisms lead to poor results of immune checkpoint inhibitors. Better understanding of these immunosuppressive mechanisms and how to counteract or suppress them has the potential to improve treatment response in prostate cancer. This will require evaluation of the tumor microenvironment and other aspects of heterogeneity, such as the immune environment, metabolism, and hypoxia, in prostate cancer. Multifaceted measures of tumor heterogeneity along with computational models to capture the interactions that drive tumor response to treatment are needed.

Oncogenes make strong therapeutic targets. Through mutation, upregulation, or gene fusion, oncogenes drive constitutive activation of signaling pathways and processes that lead to tumor development and progression. Shutting off the signaling caused by the dysfunctional oncogene is the goal and this often means developing a small molecule drug to disrupt or turn off the oncogene. However, when a tumor suppressor is lost, the therapeutic strategy is not as straightforward. Reactivation or upregulation of a non-mutant allele of a tumor suppressor, such as TP53, is a strategy that has proven difficult to effectively implement. Finding alternative ways to leverage the identified vulnerability, such as synthetic lethality, can be successful. For example, the synthetic lethal interaction between PARP inhibition and homologous recombination-deficient tumors has been successful in the clinic (103). However, determining an effective therapeutic strategy to target tumor suppressors remains a fundamental challenge in cancer biology. In prostate cancer, as we described above, the challenge is amplified by the fact that the disease is often a result of the loss of multiple tumor suppressors (17, 33). New modeling approaches must be considered to account for the loss of multiple tumor suppressors, while also considering androgen signaling.

Finally, prostate cancer researchers recognize the unique challenges that culturing primary prostate cancer cells represents. The commonly used, tumor-derived and immortalized prostate cancer cell lines are limited when compared to other cancer types such as breast and lung cancer (104, 105). In addition, the prostate cancer cell lines poorly represent the genomic diversity and loss of tumor suppressors that were previously described. Many of the cell lines that are in common use today were derived from metastasis and likely do not model the primary disease faithfully (106). Fundamentally, prostate cancer is defined by tissue pathology. Cell lines lack appropriate tissue morphology so cannot represent pathological diversity. Mouse models are available that provide evaluation of pathology and molecular modeling, but they remain expensive and technically challenging when more than two or three molecular alterations are modeled. Prostate cancer organoids are a newer model system with the organoids retaining histological and molecular features of the patient tumors from which they were derived (107, 108). They have been used to model a wider range, including rare forms, of prostate cancer (109), along with providing a platform for pharmacological testing (110, 111). However, they remain a challenge to culture with poor overall success rates (112). Tissue recombination, mixing mesenchyme with epithelium, allows for modeling components of the tumor stroma with tumor cells or tumor initiating cells. Tissue recombination is a powerful model that has the potential to dissect contributions of both the epithelium and stroma in tumorigenesis, aggressiveness, and tissue pathology. Tissue recombinant models have been developed for human, mouse and rat. Mouse stem cell lines can be genetically engineered *in vitro* with multiple drivers and modeled *in vivo* by tissue recombination (113, 114, 115, 116). Mouse syngeneic models have the added advantage of the ability to model how epithelium and stroma affect the immune microenvironment. To date, tissue recombinant models are underutilized, primarily due to technical challenges that limit the number of laboratories worldwide with the expertise in the models.

## Opportunities for systems biology to advance prostate cancer research in the future

**Figure 3:**
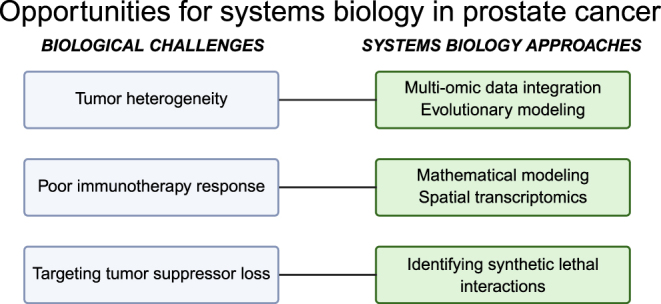
Biological challenges in prostate cancer research and the associated systems biology modeling approaches that can be used to address those challenges.

Cancer systems biology approaches provide a comprehensive, data-driven framework to study and model multi-scale complexities of cancer. Many cancer types, including breast, lung, colorectal, and melanoma, have benefited from these systems-level approaches, and the successes in these cancer types offer templates or paths that can be applied to advancing prostate cancer research. These approaches can help bridge the gap between prostate cancer pathology and the molecular features driving the disease, evaluate immunotherapy potential, identify bypass mechanisms in androgen signaling, predict when treatment resistance might arise, and link these models with clinical data to better understand prostate cancer development, progression and treatment response (Fig. 3).

In breast cancer, molecular subtypes are used to identify therapeutic options for patients (117, 118, 119). By integrating multi-omics data, researchers have refined the classification of breast cancer into distinct subtypes, enabling more precise and personalized therapeutic options. Systems approaches have also identified mechanisms of resistance to therapies such as HER2 inhibitors and hormone therapies, facilitating the development of combination treatments. As discussed, associations between pathology and molecular drivers are complicated by tumor heterogeneity and the multi-hit nature of prostate cancer. Advancing our understanding of the molecular features that drive aggressive disease development will likely require integrating data on SNVs, protein expression, and other molecular features, along with histopathological characteristics of prostate tumors. This more comprehensive modeling of prostate cancer has the potential to better identify patients responsive to standard treatments and identify novel treatment options.

Systems approaches have been used to model the tumor microenvironment and identify cellular patterns related to immunotherapy response (120). Despite limited success with immune checkpoint inhibitors in prostate cancer, novel tools, technologies, and systems biology modeling can be used to study the immune microenvironment in prostate cancer, potentially helping to identify the immunosuppressive mechanisms that limit therapeutic efficacy (102). Spatial transcriptome and proteome technologies are being used to define the cell types, signaling patterns, and spatial organization of the tumor microenvironment in immune checkpoint responsive and resistant tumors (121), leading to new insights into how resistant tumors evolve. For example, B and T cell signatures associated with acquired resistance have been defined (122), and the amount of tumor-associated macrophages and gene expression programs that drive this upregulation has been associated with immunotherapy resistance (123). There are many other factors that play into immunotherapy response, including non-immune cell stromal content (e.g., cancer associated fibroblasts), tumor mutation burden, microsatellite instability, and checkpoint target protein expression data (124, 125, 126). Leveraging this information, along with the spatial transcriptome and proteome data, tumor microenvironment models of interactions between tumor cells and the tumor microenvironment can be built in prostate cancer to identify potential vulnerabilities for immunotherapies (127, 128, 129, 130).

As discussed, an active area of prostate cancer research is to determine the bypass mechanisms of androgen receptor signaling that inevitably arise when therapeutically targeted. Signaling pathways have been extensively modeled across cancer types (131). Systems approaches can similarly be used in prostate cancer to model and predict how AR mutations, AR amplifications, AR variants, or alternative signaling pathways facilitate therapy resistance. By modeling these networks, novel therapeutic targets could be identified that are aimed at overcoming resistance to ADT and other androgen receptor signaling inhibitors. Similar to the work by West *et al.* (68, 74), these targets could then be tested *in silico* to evaluate the potential benefits of single drug or drug combinations. Extensive follow-up work would be required to determine the viability of such novel treatments, but the potential exists for advancing systems-level models in this space.

Looking forward, cancer systems biology approaches hold promise in helping to advance prostate cancer research by providing a more integrated and holistic view of the disease. These approaches can be used to address the many challenges in understanding the link between genetics and pathology, and identify modes of treatment resistance and response, all with the goal of improving outcomes and survival rates for prostate cancer patients.

## Declaration of interest

LG receives research funding to her institution from Pfizer, Johnson & Johnson, and Merck. All authors drafted and edited the manuscript.

## Funding

This work is partially supported by NCIhttps://doi.org/10.13039/100000054 grants K12CA086913 to LSG, CA262279 to SDC and CA231978 to SDC and JCC.
